# Mycobacterium ulcerans Population Genomics To Inform on the Spread of Buruli Ulcer across Central Africa

**DOI:** 10.1128/mSphere.00472-18

**Published:** 2019-02-06

**Authors:** Koen Vandelannoote, Delphin Mavinga Phanzu, Kapay Kibadi, Miriam Eddyani, Conor J. Meehan, Kurt Jordaens, Herwig Leirs, Françoise Portaels, Timothy P. Stinear, Simon R. Harris, Bouke C. de Jong

**Affiliations:** a Department of Biomedical Sciences, Institute of Tropical Medicine, Antwerp, Belgium; b Evolutionary Ecology Group, University of Antwerp, Antwerp, Belgium; c Institut Médical Evangélique, Kimpese, Democratic Republic of Congo; d Institut National de Recherche Biomédicale, Kinshasa, Democratic Republic of Congo; e Invertebrates Section, Royal Museum for Central Africa, Tervuren, Belgium; f Department of Microbiology and Immunology, University of Melbourne, Melbourne, Victoria, Australia; g Wellcome Trust Sanger Institute, Cambridge, United Kingdom; U.S. Centers for Disease Control and Prevention

**Keywords:** bacterial pathogen transmission, Buruli ulcer, Democratic Republic of the Congo, microbial comparative population genomics, molecular evolution, phylogeography

## Abstract

Buruli ulcer is a neglected tropical disease of skin and subcutaneous tissue caused by infection with the pathogen Mycobacterium ulcerans. Many critical issues for disease control, such as understanding the mode of transmission and identifying source reservoirs of M. ulcerans, are still largely unknown. Here, we used genomics to reconstruct in detail the evolutionary trajectory and dynamics of M. ulcerans populations at a central African scale and at smaller geographical village scales. Whole-genome sequencing (WGS) data were analyzed from 179 M. ulcerans strains isolated from all Buruli ulcer foci in the Democratic Republic of the Congo, The Republic of Congo, and Angola that have ever yielded positive M. ulcerans cultures. We used both temporal associations and the study of the mycobacterial demographic history to estimate the contribution of humans as a reservoir in Buruli ulcer transmission. Our phylogeographic analysis revealed one almost exclusively predominant sublineage of M. ulcerans that arose in Central Africa and proliferated in its different regions of endemicity during the Age of Discovery. We observed how the best sampled endemic hot spot, the Songololo territory, became an area of endemicity while the region was being colonized by Belgium (1880s). We furthermore identified temporal parallels between the observed past population fluxes of M. ulcerans from the Songololo territory and the timing of health policy changes toward control of the Buruli ulcer epidemic in that region. These findings suggest that an intervention based on detecting and treating human cases in an area of endemicity might be sufficient to break disease transmission chains, irrespective of other reservoirs of the bacterium.

**IMPORTANCE** Buruli ulcer is a destructive skin and soft tissue infection caused by Mycobacterium ulcerans. The disease is characterized by progressive skin ulceration, which can lead to permanent disfigurement and long-term disability. Currently, the major hurdles facing disease control are incomplete understandings of both the mode of transmission and environmental reservoirs of M. ulcerans. As decades of spasmodic environmental sampling surveys have not brought us much closer to overcoming these hurdles, the Buruli ulcer research community has recently switched to using comparative genomics. The significance of our research is in how we used both temporal associations and the study of the mycobacterial demographic history to estimate the contribution of humans as a reservoir in Buruli ulcer transmission. Our approach shows that it might be possible to use bacterial population genomics to assess the impact of health interventions, providing valuable feedback for managers of disease control programs in areas where health surveillance infrastructure is poor.

## Abstract (precis)

Buruli ulcer is a destructive skin and soft tissue infection caused by Mycobacterium ulcerans. The disease is characterized by progressive skin ulceration, which can lead to permanent disfigurement and long-term disability. Currently, the major hurdles facing disease control are incomplete understandings of both the mode of transmission and environmental reservoirs of M. ulcerans. As decades of spasmodic environmental sampling surveys have not brought us much closer to overcoming these hurdles, the Buruli ulcer research community has recently switched to using comparative genomics. The significance of our research is in how we used both temporal associations and the study of the mycobacterial demographic history to estimate the contribution of humans as a reservoir in Buruli ulcer transmission. Our approach shows that it might be possible to use bacterial population genomics to assess the impact of health interventions, providing valuable feedback for managers of disease control programs in areas where health surveillance infrastructure is poor.

## INTRODUCTION

Mycobacterium ulcerans causes a slowly progressing necrotizing infection of skin and soft tissue known as Buruli ulcer (BU) disease (1). In BU patients, early diagnosis followed by 8 weeks of treatment with a combined antibiotic regimen (rifampin and streptomycin-clarithromycin) is key to preventing complications that can arise from severe skin ulcerations (2). BU is a neglected tropical disease that can exceed the incidence of leprosy and tuberculosis in some areas of high endemicity (3). The disease has been reported in more than 30 countries worldwide; however, the biggest burden of disease is still found in impoverished rural areas of West and Central Africa (4), where 1,750 new cases were notified to the WHO in 2017 (5).

BU epidemiology is characterized by its patchy focal distribution within countries were it is endemic (4). Disease foci are known to primarily occur around low-lying marshes, wetlands, and riverine areas (3). As living or working close to these slow-flowing or stagnant water bodies is a known risk factor for M. ulcerans infection (6), and as human-to-human transmission is very rare, it is generally believed that M. ulcerans is an environmental mycobacterium that can infect humans through introduction via microtraumata to the skin (7). However, the exact mode of disease transmission and the environmental reservoir(s) of M. ulcerans remain enigmatic in Africa (8), as culturing the slow-growing mycobacterium from nonclinical environmental sources has proved to be particularly challenging (9). This has severely hampered the ability of the BU community to establish the presence of viable M. ulcerans in potential environmental reservoirs.

As M. ulcerans has the genome signature of a “niche-adapted” mycobacterium, it is considered unlikely to be found free living in various aquatic or terrestrial environments and is rather more likely living in close association with a host organism (10). We recently observed a temporal association between humans and the spread of BU across Africa during the period of neoimperialism (late 19th to early 20th century) (11). The introduction of both lineage Mu_A2 in the continent and lineage Mu_A1 in three well-sampled disease foci coincided closely with the instigation of colonial rule. Since these disease foci were inhabited prior to the arrival of the European powers and since introduction only occurred after colonization, we posited that displaced humans with actively infected openly discharging BU lesions inadvertently contaminated aquatic environments during water contact activities and thus spread the mycobacterium.

Conventional genetic fingerprinting methods have largely failed to differentiate clinical disease isolates of M. ulcerans (12), leading to their replacement with whole-genome sequencing (WGS) (11, 13–16). The greater resolution offered by genomics to discriminate between isolates, combined with the availability of novel state-of-the-art demographic models in Bayesian phylogenetic analysis (17, 18), is opening up new possibilities to explore the pathogen’s cryptic epidemiology and disease ecology. A recent study in southeastern Australia (15) identified a striking relationship between the number of Victorian BU cases through time and the mycobacterial demographic history inferred from the genomic data. As such, modeling the demographic dynamics indicated the amount of BU cases was likely to be influenced by the abundance of the pathogen, providing an explanation for the apparent recent rise of Victorian cases. Likewise, a study on Mycobacterium tuberculosis used similar comparative genomics to investigate both the mycobacterial historical demography and the timeline of acquisition of antimicrobial resistance during a major outbreak of drug-resistant tuberculosis (TB) in Buenos Aires, Argentina (19). The work indicated that a multidrug-resistant M. tuberculosis (MDR-TB) strain had been circulating for 15 years before its outbreak was detected. Furthermore, modeling of the past mycobacterial demography indicated a rapid increase in the mycobacterial population size in the early 1990s during a steep upsurge of HIV-related MDR-TB.

The present study focuses on endemic BU foci in the Democratic Republic of the Congo (DRC) and some of its neighboring countries. Prior to 2002, BU control in the Democratic Republic of the Congo suffered from decades of neglect and conflict, affecting the vast nation’s health and sanitation infrastructure (4). The first BU case of the Democratic Republic of the Congo was reported in 1950, in the Kwilu province (20). Since this first description, microbiologically confirmed cases have been identified in the provinces of Equateur, Haut-Uele, Ituri, Kwango, Kwilu, Kongo Central, Mai-Ndombe, and Maniema (see Fig. S1 in the supplemental material) (21). The main focus of BU endemicity in the country is located in the Songololo territory of the Kongo Central province and encompasses the areas of high endemicity in the rural health zones of Kimpese and Nsona-Mpangu. The population of the territory (estimated at around 154,000 inhabitants) leads a sedentary lifestyle and lives mostly from subsistence agriculture and (petty) trade. Since no epidemiological studies were conducted in the territory until the 1960s and 1970s (22), it remained unclear whether BU was newly introduced or an old, undetected, and expanding illness in the region. In the aftermath of the Angolan civil war (1975 to 2002), BU was frequently diagnosed in Angolan refugees who lived in refugee camps located in the Songololo territory. As cases have been reported in Angola (23), the possibility has been put forward (24) that these patients were infected in Angola and reintroduced BU in the region.

10.1128/mSphere.00472-18.1 FIG S1 BU distribution in the Democratic Republic of the Congo as established in 2013. Selected health zones are highlighted red (area of endemicity) and yellow (suspected area of endemicity) based on all studies, surveys, and activity reports published between 1950 and 2013 (21). The administrative borders of the provinces of the Democratic Republic of the Congo were obtained from the Référentiel Géographique Commun (the Democratic Republic of the Congo). The river layer was translated from the river-surface water body network data set of the African Water Resource Database of FAO. Download FIG S1, JPG file, 2.1 MB. Copyright © 2019 Vandelannoote et al. 2019 Vandelannoote et al. This content is distributed under the terms of the Creative Commons Attribution 4.0 International license.

We believe a better understanding of the transmission and the disease dynamics of M. ulcerans infection could have a direct impact on the development of effective and appropriate control strategies against the disease. In this study, we sequenced and compared the genomes of 179 M. ulcerans strains isolated from patients in the Democratic Republic of the Congo, The Republic of the Congo (RC), and Angola over a 52-year period to investigate the microevolution and population dynamics of this pathogen during its establishment in this specific region.

## RESULTS

### Genome sequence comparisons of 179 M. ulcerans isolates from Central Africa.

To understand the dynamics and timing of the spread of M. ulcerans across Central Africa, we sequenced the genomes of 179 clinical isolates that were obtained between 1962 and 2014 and spanned most of the known areas of BU endemicity in the Democratic Republic of the Congo, The Republic of the Congo, and Angola (see Table S1 in the supplemental material). To prevent mapping the obtained sequence reads to a reference that diverged significantly from these isolates, we assembled a new, complete closed DRC M. ulcerans reference chromosome using PacBio reads. This reference chromosome received the strain name SGL03 (for Songololo territory 2003). SGL03 comprises a single 5,625,184-bp (6,422 bp smaller than the Ghanaian reference chromosome Agy99) circular bacterial chromosome with a G+C content of 65.5%. Whole-genome comparisons between SGL03 and Agy99 revealed extensive synteny and collinearity. However, a total of 12 large (>100 bp) indels were identified between SGL03 and Agy99 (see Table S2). Most indel events were mediated by copies of insertion (IS) elements IS*2404* and IS*2606*; these either flanked deletions or they were present in the deleted or substituted sequence stretches. Well represented in the deleted sequences were pseudogenes that either contained frameshift mutations or were disrupted by IS elements.

10.1128/mSphere.00472-18.7 TABLE S1 Strain information and basic sequencing statistics. Download Table S1, XLSX file, 0.02 MB. Copyright © 2019 Vandelannoote et al. 2019 Vandelannoote et al. This content is distributed under the terms of the Creative Commons Attribution 4.0 International license.

10.1128/mSphere.00472-18.8 TABLE S2 Large (>100-bp) genomic indels between M. ulcerans reference chromosomes SGL03 (DRC) and Agy99 (Ghana). Download Table S2, XLSX file, 0.01 MB. Copyright © 2019 Vandelannoote et al. 2019 Vandelannoote et al. This content is distributed under the terms of the Creative Commons Attribution 4.0 International license.

Illumina sequence reads of the sample panel were aligned to the newly assembled SGL03 chromosome and, after removing any diversity detected in repetitive IS elements and ignoring small indel polymorphisms, we found 6,655 single nucleotide polymorphisms (SNPs) uniformly distributed along the bacterial chromosome, which amounts to 1 SNP per 846 bp (see Fig. S2). A total of 161 clones (unique genomes) were discerned among the isolate panel.

10.1128/mSphere.00472-18.2 FIG S2 Distribution of SNPs identified in the entire sequenced sample panel (Mu_A1 and Mu_A2) compared to the Congolese M. ulcerans SNG03 reference genome. The *y* axis corresponds to SNP counts per 10,000-bp window, the dashed line indicates the average rate of 11.8 SNPs per 10,000 bp (or 1 SNP per 846-bp window). Download FIG S2, TIF file, 1 MB. Copyright © 2019 Vandelannoote et al. 2019 Vandelannoote et al. This content is distributed under the terms of the Creative Commons Attribution 4.0 International license.

**FIG 1:**
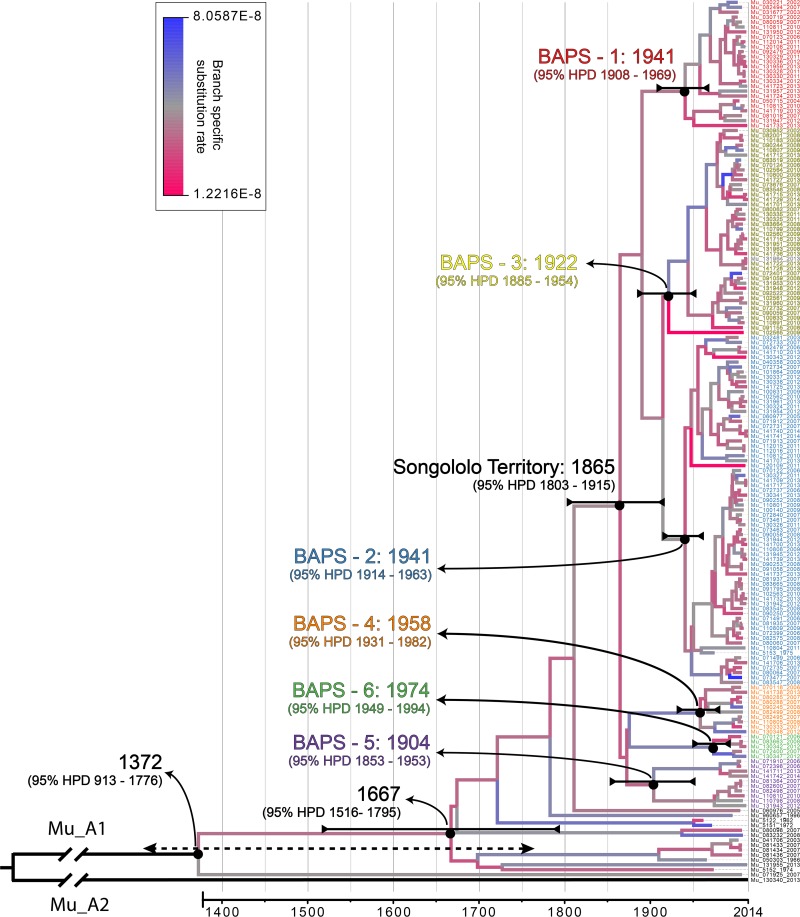
Bayesian maximum clade credibility phylogeny for DRC, RC, and Angolan M. ulcerans isolates built with the 179 isolates. The tree was visualized and colored in Figtree v1.4.2. Branches are color coded according to their branch-specific substitution rate (legend at top). Branches defining major lineages are annotated on the tree. Tip labels of Songololo territory isolates are color coded according to their respective BAPS groups. Divergence dates (mean estimates and their respective 95% HDPs) are indicated in black for major nodes. Note that 95% HDP intervals grow larger closer to the root of the tree, as increasingly less timing calibration information (from tip dates) is available the further one goes back in time.

A Bayesian time-measured phylogeny was inferred from a whole-genome alignment of the isolates (Fig. 1). Both known lineages of African M. ulcerans were identified within the Central African isolate panel: 178/179 (99.4%) corresponded to lineage Mu_A1 and 1/179 (0.6%) corresponded to the uncommon lineage Mu_A2. The average pairwise SNP difference (SNPΔ) between Mu_A1 Central African isolates was low (59 SNPs, standard deviation [SD] = 42), as the majority of the discovered diversity derived from the relatively large genetic distance (5,270 SNPs, SD = 7) between Mu_A1 and the single Mu_A2 isolate from the region. The Mu_A2 isolate (ITM130340) originated from a patient (female [F], 40 years old) from the hamlet Kilima in the Songololo territory (Nkamuna health area) (see Fig. S4). We were unable to retrospectively interview the patient to identify any travel history or activity that could explain the unexpected Mu_A2 distribution.

### Phylogenetic analysis reveals strong geographical restrictions on M. ulcerans dispersal at high-level geographical scales.

**FIG 2:**
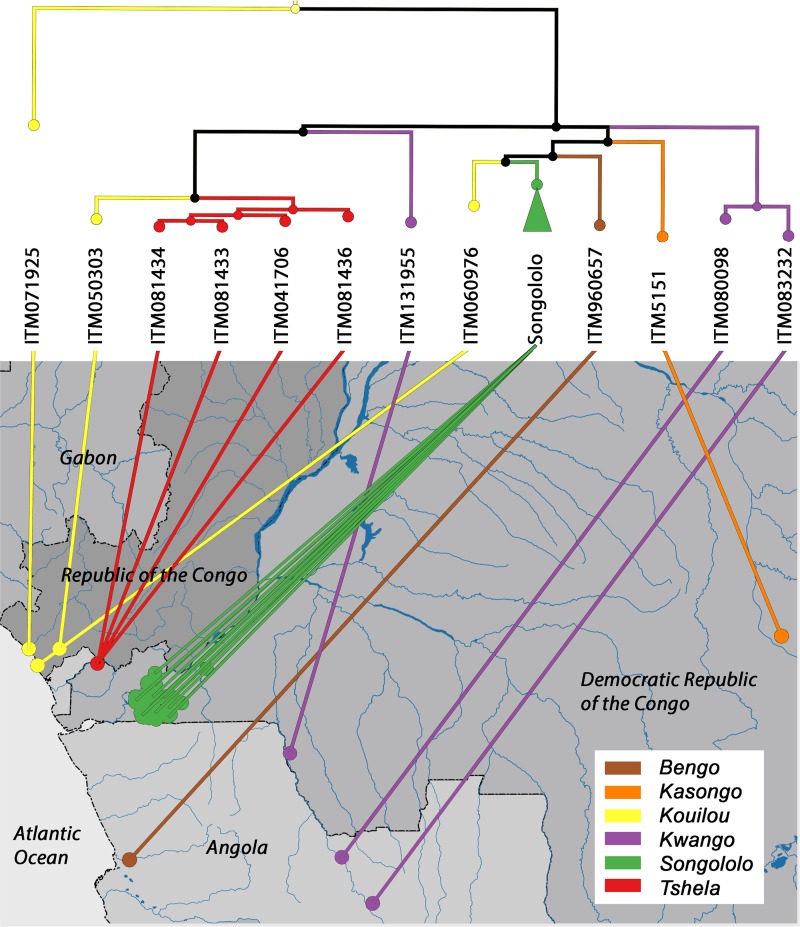
Phylogeography of DRC, RC, and Angolan M. ulcerans isolates. A maximum likelihood phylogeny is drawn for lineage Mu_A1 with branches color coded according to BU disease focus (legend bottom right). The ML phylogeny is based on 1,373 SNP differences detected across the whole core genome of 135 sequenced isolates with GPS data. Nodes in the tree with bootstrap support below a set threshold of 70% were collapsed to polytomies while preserving the length of the tree. The green clade formed by 123 isolates from the Songololo territory disease focus is collapsed in the tree. The tips of the tree are connected to the residence locations of patients from whom the strains were isolated. The administrative borders of countries were obtained from the Global Administrative Unit Layers data set of FAO. The river layer was translated from the river-surface water body network data set of the African Water Resource Database of FAO.

Within an M. ulcerans phylogeny of the entire African continent (see Fig. S3), the single Songololo Mu_A2 isolate clustered together with a clade of 8 other Mu_A2 isolates originating from Benin, Gabon, and Cameroon. Furthermore, a distinct Mu_A1 isolate from The Republic of the Congo (ITM_071925) clustered together with a small clade of Nigerian and Cameroonian M. ulcerans isolates. More importantly, however, all other 177 Mu_A1 isolates of the Central African panel formed a monophyletic group within that continental African phylogeny. There was distinct spatial clustering of M. ulcerans from the different endemic BU foci within the phylogeny. For instance, all 123 isolates of the endemic BU focus of the Songololo territory formed a strongly supported monophyletic group (Fig. 2). The Songololo territory isolates had an average pairwise SNPΔ of 46 (SD = 18) and were unrelated to the four isolates from the neighboring Tshela territory (northwest in the Kongo Central province) that formed a separate monophyletic group (Fig. 2). We cannot identify a specific historic geographical route that these bacterial lineages followed, but the phylogenetic evidence clearly links these separate clonal expansions as a single epidemic.

10.1128/mSphere.00472-18.3 FIG S3 The position of Central African isolates in the continental African M. ulcerans tree. Bayesian maximum clade credibility phylogeny drawn for lineage Mu_A1 and Mu_A2 for the 179 isolates sequenced in this study plus 144 African M. ulcerans isolates sequenced previously (11) (total *n* = 323). DRC, RC, and Angolan isolates are highlighted in pink in the phylogeny. Branches defining major lineages are annotated on the tree. Download FIG S3, JPG file, 2.5 MB. Copyright © 2019 Vandelannoote et al. 2019 Vandelannoote et al. This content is distributed under the terms of the Creative Commons Attribution 4.0 International license.

### The clustering of M. ulcerans genotypes ends at fine geographical scales.

**FIG 3:**
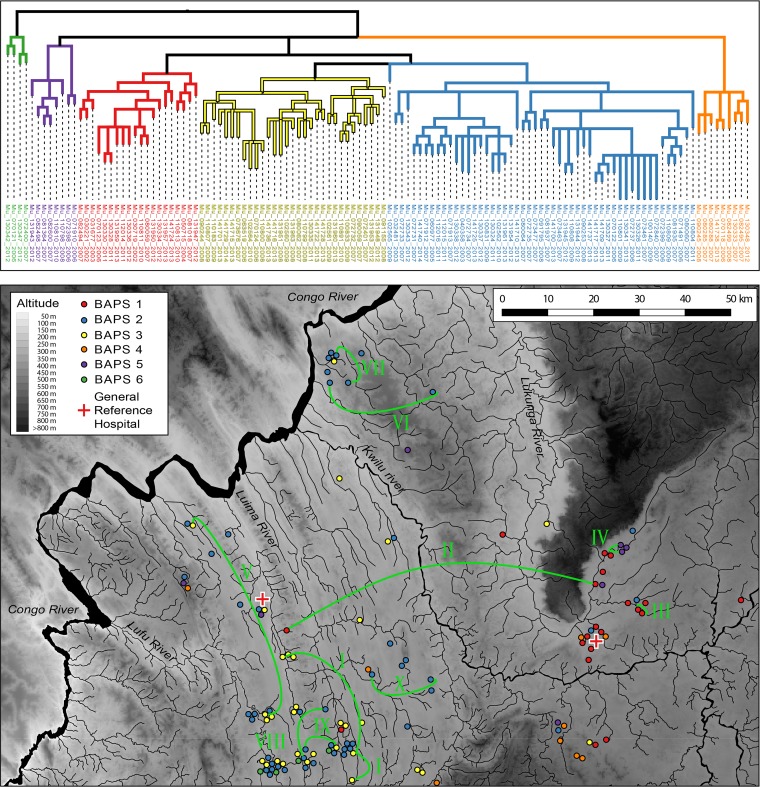
Phylogeography of the Songololo territory BU disease focus. A maximum likelihood phylogeny is drawn for lineage Mu_A1. The ML phylogeny is based on 684 SNP differences detected across the whole core genome of 123 sequenced isolates from the Songololo territory with GPS data. Branches are color coded according to their respective BAPS groups as indicated in the legend (the best-visited BAPS partitioning scheme of our sample yielded a natural log marginal likelihood of −9941.8805). Nodes in the tree with bootstrap support below a set threshold of 70% were collapsed to polytomies while preserving the length of the tree. The residence locations of patients from whom the isolates were grown are colored according to the BAPS groups the corresponding isolates belonged to. Identical genomes identified in different patients are interconnected by the green curves, which are annotated with Roman numerals. The background map was created using elevation data from the Shuttle Radar Topography Mission (SRTM). The river layer (Congo River and its tributaries) was digitized from declassified Soviet military topographic maps xb33-13, xb33-14, xb33-15, xb33-16, and xb33-17 (scale 1:200,000) and xb33-1 (scale 1:500,000).

We then explored the geographical distribution of M. ulcerans genotypes at a finer geographical scale: that of the Songololo territory. The 123 Songololo isolates originating from 123 individual patients were spread evenly over the territory, and the majority of health areas with a “modest” to “high” BU burden were well represented (Fig. S4). Bayesian model-based inference of the genetic population structure revealed the existence of six groups (designated BAPS groups 1 to 6) within the territory (Fig. 3). The six groups generally cooccurred, as in some regions of the territory, multiple groups were found to be circulating simultaneously. In the health area of Lovo for instance, up to five different groups were cocirculating (BAPS 1 to 5). The groups were, however, distributed differently over the study region: groups 2, 4, and 5 were found widely dispersed, while groups 1, 3, and 6 were more restricted (Fig. 3). Group 1 (*n* = 20) was found almost exclusively in the eastern Kimpese health area, while group 3 (*n* = 31) was localized in the western Nsona-Pangu health area (see Fig. S5). Group 6 was uncommon (*n* = 4) and found solely in the southwest. Within groups, there were some distinct subgroups, which very occasionally also had a limited distribution across the region. For example, one specific subgroup of BAPS group 2 consisted of seven isolates that all originated from a 90-km^2^ zone covering the neighboring health areas of Mukimbungu and Kasi (Fig. S5). However, other subgroups were far more broadly distributed, with the extreme example of identical genomes identified in different BU patients separated by larger distances (Fig. 3, I to X). A total of ten such genomes that were identified multiple times in the Songololo territory were discerned (Table 1). The average geographical distance between the domiciles of patients identified with isolates with identical genomes was 17.3 ± 18.1 km.

**TABLE 1:** Identical genomes identified in different BU patients of the Songololo Territory

Identical genome	Identification no. for isolate (YOI):^*a*^			Geographical distance (km)	No. of yrs between isolation dates
	1	2	3		
I	ITM102560 (2009)	ITM131951 (2008)	ITM141716 (2013)	21.1, 25.6, 5.7	1, 5, 4
II	ITM130328 (2011)	ITM130330 (2011)		56.5	0
III	ITM130336 (2012)	ITM131959 (2013)		0.0	1
IV	ITM081364 (2007)	ITM082600 (2007)		0.1	0
V	ITM141715 (2013)	ITM141729 (2014)		37.2	1
VI	ITM072731 (2007)	ITM141740 (2014)		18.7	7
VII	ITM112015 (2011)	ITM112016 (2011)		6.0	0
VIII	ITM073463 (2007)	ITM141700 (2013)		11.2	6
IX	ITM081935 (2007)	ITM110809 (2009)		5.7	2
X	ITM141709 (2013)	ITM141717 (2013)		11.4	0

a YOI, year of isolation.

10.1128/mSphere.00472-18.4 FIG S4 Sampling effort and the distribution of the BU disease burden in the health zones and health areas of the Kongo Central province of the Democratic Republic of the Congo. The distribution of all BU cases per health zone (top) or health area (bottom) was determined from all disease notifications reported since the start of the national BU program (PNLUB) in 2002 until 2014. The red crosses denote the General Reference Hospitals of the Institut Médical Evangélique (IME) in Kimpese and that of Nsona-Mpangu. Yellow points represent the residences of BU patients from whom M. ulcerans disease isolates were grown at the time of clinical visit. A 2008 study (28) estimated the overall BU prevalence of the Songololo territory at 3.3/1,000 population, with considerable variation (0.0 to 27.5/1,000 population) among the 40 health areas of the territory. Note the health areas of CBCO, CECO, Kimbala, Kimbanguist, and Yanga Dia Songa are depicted here as the merged delimitation of Kimpese city. Download FIG S4, JPG file, 2.6 MB. Copyright © 2019 Vandelannoote et al. 2019 Vandelannoote et al. This content is distributed under the terms of the Creative Commons Attribution 4.0 International license.

10.1128/mSphere.00472-18.5 FIG S5 Detailed view of the phylogeography of BAPS clusters 1 (eastern) and 3 (western) and subcluster Mukimbungu-Kasi of the Songololo territory BU disease focus. For clarity, not all connecting lines are plotted. The distribution of isolates and the overlaying of the phylogenetic tree was performed with GenGIS v2.5.0 (42), based on the household GPS coordinates of each patient and the whole-genome ML phylogeny (same as in Fig. 3) of their corresponding M. ulcerans isolates. Download FIG S5, JPG file, 1.6 MB. Copyright © 2019 Vandelannoote et al. 2019 Vandelannoote et al. This content is distributed under the terms of the Creative Commons Attribution 4.0 International license.

### The Central African mutation rate of M. ulcerans is similar to that inferred on a continental scale.

We derived a timed phylogeny of Central African M. ulcerans while simultaneously inferring mutation rates and dates of divergence of key M. ulcerans clades (Fig. 1). In this process, a molecular clock was estimated using correlations between phylogenetic divergence and isolation times of heterochronous disease isolates. As a result, a mean genome wide substitution rate of 4.38E−8 per site per year (95% highest posterior density [HPD] interval, 2.83E−8 to 6.03E−8]) was demonstrated, which corresponds to an accumulation rate of 0.23 SNPs per bacterial chromosome per year (95% HPD interval, 0.15 to 0.32).

The Bayesian phylogenetic analysis indicates that lineage Mu_A1 had been introduced in Central Africa multiple hundreds of years ago (*t*_MCRA_ [Mu_A1], 1372; 95% HPD, 913 to 1776), while the timing of the BU introduction event in the Songololo territory was estimated at around 1865 (95% HPD, 1803 to 1915) (Fig. 1). Finally, the time tree also indicates that the separated “eastern” (*t*_MCRA_ [BAPS-1], 1941; 95% HPD, 1908 to 1969) and “western” (*t*_MCRA_ [BAPS-3], 1922; 95% HPD, 1885 to 1954) Songololo groups have most likely remained segregated over a timespan of half a century.

### Demographic history of M. ulcerans in the Songololo territory.

**FIG 4:**
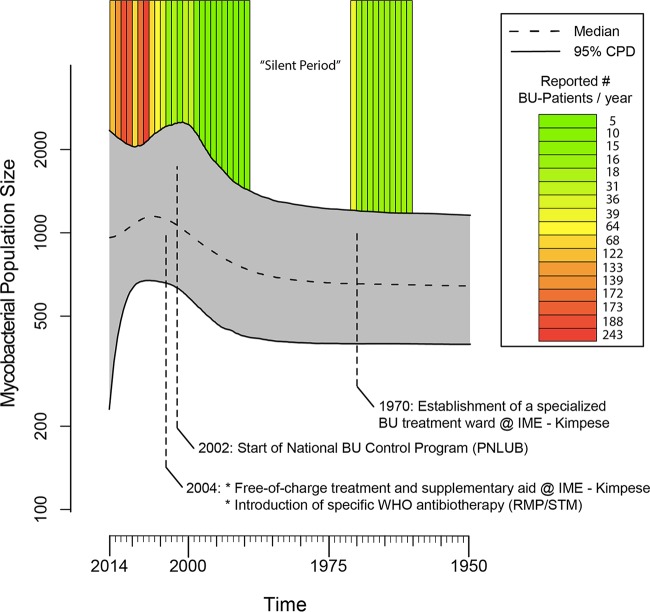
The demographic history of M. ulcerans in the Songololo territory and the annual amount of cases from the Territory reported by the national BU program (Program National de Lutte contre L’Ulcère de Buruli [PNLUB]). The extended Bayesian skyline plot displays a relative measure of the mycobacterial population size (N_e*_ τ) through time (with N_e_ representing the effective population size and τ symbolizing the mean mycobacterial generation time). As this is an arbitrary scale, it only allows us to discuss relative increases or decreases to the population size. The central dotted line represents the median mycobacterial population size with its 95% central posterior density (CPD) interval represented by the upper and lower lines. Note the *y* axis is on the log scale. New BU cases were regularly identified before 1970, after which there was a 20-year-long “silent” period in the scientific literature, during which no cases were reported. During this period, the hospital lost the majority of its specialized personnel, which was partially due to the political situation in the Democratic Republic of the Congo at that time. This led to Institut Médical Evangélique’s (IME’s) lowest recorded (all-cause) admission rate of 4.5 patients/year between 1989 and 1999 (58). Later, in 2002, the national BU program PNLUB was started, and during 2002 to 2004, an apparent resurgence of BU was reported in the Songololo territory (59). Since the end of 2004, the IME hospital in Kimpese launched a specialized BU program, offering free-of-charge treatment and supplementary aid. Additionally, starting in 2004, patients benefited from specific antibiotherapy which was introduced in accordance with WHO recommendations (60). Since the start of the BU control project, a strong increase was noted in the number of notified BU cases, including those admitted to IME hospital (61).

The reconstruction of the demographic history of M. ulcerans in the Songololo territory involved the coestimation of its time tree, the mycobacterial population size at different points along the timescale of that phylogeny, and all other parameters of the employed model of molecular evolution. Consequently, the resulting plot of the population history includes credibility intervals that represent the combined phylogenetic and coalescent uncertainties. An inspection of the extended Bayesian skyline plot (EBSP) (Fig. 4) indicated that the M. ulcerans population size remained stable until the early 1980s, after which it increased slightly during the course of the 1990s, until it reached a peak around 2004. This was followed by a small decline that persisted until 2014. We identified temporal parallels between the observed past population dynamics of M. ulcerans from the Songololo territory and the timing of health policy changes managing the BU epidemic in that region (Fig. 4), though we need to recognize overlap in credibility intervals surrounding the estimates during these periods. We checked for factors that might bias the reconstruction of the mycobacterial population size over time by conducting extensive resampling and randomization experiments (see Fig. S6).

10.1128/mSphere.00472-18.6 FIG S6 Comparison of Bayesian estimates of nucleotide substitution rates for real and reshuffled tip dates. Filled squares and circle represent mean estimates, while bars indicate values of the 95% highest probability density (HDP) interval. The estimate obtained using the real tip date associations (circle) is shown on the far right, while estimates from random associations (squares) are shown on the left. All randomized data sets were analyzed in BEAST2 using identical model settings as used in the analysis of the real tip date data. Note the *y* axis is on the log scale. Download FIG S6, TIF file, 0.8 MB. Copyright © 2019 Vandelannoote et al. 2019 Vandelannoote et al. This content is distributed under the terms of the Creative Commons Attribution 4.0 International license.

## DISCUSSION

The demographic history of a pathogen population leaves a “signature” in the genomes of modern representatives of that population (18). Reconstructing this history allows us to gain valuable insights into the processes that drove past population dynamics (25). We recognized temporal parallels between the mycobacterial population dynamics and the timing of health policy changes managing the BU epidemic in the Songololo territory (Fig. 4). The mycobacterial population size increased in the territory during a period of decreased attention to BU that resulted in the loss of specialized personnel. After the start of a national BU program and the implementation of free-of-charge treatment, a strong increase was noted in the number of admitted BU cases which concorded with a detected inflection—perhaps a small drop—of the mycobacterial population size. These observations suggest that control strategies at the public health level may have decreased the size of the human M. ulcerans reservoir and that this reservoir is important in sustaining new infections. This hypothesis predicts that even if other environmental reservoirs exist, the number of M. ulcerans infections will decrease if only human cases are treated.

The M. ulcerans phylogeography revealed one almost exclusively predominant sublineage of Mu_A1 that arose in Central Africa and proliferated in the different foci of endemicity of the Democratic Republic of the Congo, Angola, and The Republic of the Congo during the Age of Discovery (15th to 18th centuries). The principal sublineage of Mu_A1 was introduced into the Songololo territory around 1865 (95% HPD, 1803 to 1915), and over the subsequent century (1865 to 1974), it established itself and evolved in six distinct groups across the territory. This timing is consistent with in-depth interviews with former patients and observations of healed lesions that suggested that M. ulcerans infections already occurred in the Songololo territory in 1935 and probably even earlier (22). The genome-based time tree of Central African M. ulcerans thus revealed that the Songololo territory became an area of endemicity while the region was being colonized by Belgium (1880s). Early during the Belgian occupation, the Songololo territory was developed heavily to link the oceanic harbor of Matadi by rail with Kinshasa, where the Congo River becomes navigable, opening up the entire interior of the Democratic Republic of the Congo for economic exploitation (26). The Songololo territory was already inhabited long before the arrival of the European colonizers. The Kongo people are believed to have settled at the mouth of the Congo River before 500 BCE (before the Common Era), as part of the larger Bantu migration (27). However, our data reveal that it was only after the start of colonial rule that the epidemic Songololo M. ulcerans clone was introduced, possibly through the arrival of displaced BU-infected humans or as a consequence of the substantial environmental changes brought by the Belgian occupation of the Songololo territory.

Similarly to recent studies that used comparative genomics to investigate the microevolution of M. ulcerans during its establishment in a continent (11) or region (13, 16), the genotypes in Central Africa show strong spatial segregation (Fig. 2). This was illustrated by the regional clustering of M. ulcerans from the different endemic BU foci (e.g., Songololo and Tshela) within the phylogenies. This clustering of cases within foci of endemicity reflects a common source of infection within the disease focus. These repeated findings indicate that when M. ulcerans is introduced in a particular area, it remains isolated, resulting in a localized clonal expansion associated with that area. An inspection of the time tree showed that a clonal complex associated with a focus of endemicity often has been pervasive in that region for a considerable time; in the case of Songololo, for around 150 years. Even within the Songololo territory, we observed that the separated eastern Kimpese health area (*t*_MCRA_ [BAPS-1], 1941) and western Nsona-Pangu health area (*t*_MCRA_ [BAPS-2], 1922) groups have most likely remained segregated over a timespan of half a century, indicating that M. ulcerans spreads relatively slowly between neighboring regions. This also indicates that environmental reservoirs of the mycobacterium in that region had to remain localized and relatively isolated.

Unlike the larger geographic scale data, at smaller geographical scales, genotypes start to co-occur. First, four Songololo BAPS groups were found to be cocirculating. Moreover, we observed completely identical genomes originating from patients living in villages separated by distances of on average 17 km, similar to the findings of recent studies (13, 14). We believe the observed “breakup” of the focal distribution pattern at smaller geographical scales can be explained by the determined low substitution rate that corresponds to the accumulation of just 0.23 SNPs per bacterial chromosome per year. The slow substitution rate severely limits the accumulation of point mutations and as such, lowers the resolving power of the genomes. This explains why (in the most extreme case), over a period of 5 years, identical genomes were discovered in three patients who lived in three different villages separated by 26 km: insufficient time has elapsed for point mutations to accumulate.

Finally, an old debate relating to the role of Angolan refugees on a resurge of BU in the Songololo territory (24) can be settled. As most of these refugees had already lived in the Democratic Republic of the Congo for several years before their diagnosis, and as some young Angolan BU patients were even born in the Democratic Republic of the Congo without even having visited Angola, an introduction from Angola was already believed to be unlikely. Now, an analysis of our phylogenies shows that no typical Angolan genotypes were detected in Songololo, indicating that the refugees were in all likelihood infected in the Democratic Republic of the Congo.

In conclusion, in the present study, we used both temporal associations and the study of the mycobacterial demographic history in a focus of endemicity to implicate human-induced changes and activities over (recent) historical scales behind the spread of BU in Central Africa. We propose that patients with infected discharging BU lesions can contaminate slow-flowing riparian and lentic environments through activities involving water contact and that these patients can constitute an important means of bacterial spread. A total of 74% of BU patients identified during a cross-sectional study (28) of the Songololo territory had ulcerative lesions (49% category I, 31% category II, and 20% category III), indicating that a high percentage of patients might be shedding bacteria into the environment and potentially indirectly infecting others. We suggest that in BU-affected areas, chains of transmission can be broken and the spread of disease stopped through improved disease surveillance, resulting in treatment during the preulcerative onset of the infection. This view is supported by the decline of BU incidence recorded in some regions of endemicity which profited from such enhanced active surveillance practices (29).

## MATERIALS AND METHODS

### Study sites.

The study covered all BU foci in the Democratic Republic of the Congo, The Republic of the Congo, and Angola that have ever yielded positive M. ulcerans cultures. The foci of endemicity of the Democratic Republic of the Congo are located in the provinces of Kwango, Kongo Central (previously known as Bas-Congo), and Maniema (see Fig. S1 in the supplemental material). The vast majority of isolates originated from the Songololo territory of the Kongo Central province. Isolates from the area of low endemicity of Tshela territory, northwest in the Kongo Central province, were also included. Isolates from the Maniema province originated from the historical BU focus of the Kasongo territory (30), which was recently assessed as still active (31). Finally, the province of Kwango was represented by a recently discovered focus of endemicity along the Kwango River, a tributary of the Congo River that forms the border between Angola and the Democratic Republic of the Congo (32) (Fig. S1).

### Bacterial isolates.

We analyzed a panel of 179 M. ulcerans strains originating from disease foci in the Democratic Republic of the Congo, The Republic of the Congo, and Angola that had been isolated between 1962 and 2014 (Table S1). As 13 isolates of this panel had no geographical information linked to them, they were included in the molecular dating work but omitted from the phylogeographical analysis. Even though the exact geographical origin of these 13 isolates was not established, we know they originated from the same hospitals as the other isolates of the panel. Based on conventional phenotypic and genotypic methods, all bacterial isolates had previously been assigned to the species M. ulcerans (33). Mycobacterial isolates were maintained for prolonged storage at ≤−70°C in Dubos broth enriched with oleic acid-albumin-dextrose (OAD) growth supplement and glycerol. In addition to the isolates sequenced here, 144 other African genomes (described in reference 11) were included to provide appropriate genetic context for interpreting the diversity and evolution of Central African M. ulcerans. Permission for the study was obtained from the ITM institutional review board (Belgium) and the ethics committee of the Public Health School of the University of Kinshasa (the Democratic Republic of the Congo).

### Sequencing.

Index-tagged paired-end sequencing-ready libraries were prepared from genomic DNA (gDNA) extracts with the Nextera XT DNA library preparation kit. Genome sequencing was performed on an Illumina HiSeq 2000 sequencer according to the manufacturers’ protocols, with 100-bp or 150-bp paired-end sequencing chemistry. Sequencing statistics are provided in Table S1. The quality of raw Illumina reads was investigated with FastQC v0.11.3 (34). Prior to further analysis, reads were cleaned with clip, a tool in the Python utility toolset Nesoni v0.130 (35). Reads were filtered to remove those containing ambiguous base calls, any reads <50 nucleotides in length, and reads containing only homopolymers. All reads were further trimmed to remove residual ligated Nextera adaptors and low-quality bases (<Q10) at the 3′ end. The average read lengths of read pairs after clipping are summarized for all isolates in Table S1.

A new, complete closed DRC M. ulcerans reference chromosome was assembled using PacBio reads. Isolate ITM032481 originated from a well-documented patient (male, 10 years old) from the hamlet Nkondo-Kiombia (Minkelo health area) (Fig. S4) who presented with a severe disseminated form of BU in 2003. BU was confirmed in the patient with all four diagnostic tests: Ziehl-Neelsen microscopy, IS*2404* qPCR, culture, and histopathology. Intact, pure high-molecular-weight gDNA was obtained from the isolate by harvesting the growth of 10 Löwenstein-Jensen (LJ) slants followed by heat inactivation (80°C for 1 h), enzymatic digestion (proteinase K, lysozyme, and RNase), and purification with the Genomic DNA buffer set (Qiagen, catalog number 19060) and 100/G Genomic-tips (Qiagen, catalog number 10243). This gDNA sample was submitted to the Duke Sequencing and Genomic Technologies Shared Resource for sequencing on a Pacific Biosciences RSII instrument. Libraries of 15 to 20 kb were constructed and sequenced on 3 SMRT cells using P5-C3 chemistry. This yielded 895 Mbp from a total of 161,629 subreads. The average subread length was 5,536 bp with a sequencing depth of 160×. Data were analyzed using SMRT Analysis v2.3.0 (Pacific Biosciences). The continuous long reads (CLR) were assembled *de novo* using the PacBio Hierarchical Genome Assembly Process 3 (HGAP.3) and polished using Quiver as previously described (36). This resulted in a single contig that was polished a final time with paired-end Illumina reads. The final contig was subsequently circularized and annotated using Prokka v1.11 (37).

The annotated closed genome was then manually curated and visualized using both Artemis v.16 (38) and Geneious v9.0.5 (39). The Congolese M. ulcerans Mu_ITM032481 bacterial reference chromosome sequence received the strain name SGL03.

### Read mapping and SNP detection.

Read mapping and SNP detection were performed using the Snippy v3.0 pipeline (40). The Burrows-Wheeler Aligner (BWA) v0.7.12 (41) was used with default parameters to map clipped read pairs to the new Congolese SGL03 reference genome. Due to the unreliability of read mapping in mobile repetitive regions, all ISE elements (IS*2404* and IS*2606*) were hard masked in these reference genomes (0.398 Mb/5.625 Mb, i.e., 7% of SGL03). After read mapping to M. ulcerans SGL03, average read depths were determined with SAMtools v1.2 (42) and are summarized for all isolates in Table S1. SNPs were subsequently identified using the variant caller FreeBayes v0.9.21 (43), with a minimum depth of 10 and a minimum variant allele proportion of 0.9. Snippy was used to pool all identified SNP positions called in at least one isolate and interrogate all isolates of the panel at that position. As such, a multiple sequence alignment of core SNPs was generated.

### Phylogeographic analysis.

Bayesian model-based inference of the genetic population structure was performed using the Clustering with linked loci module (44) in BAPS v.6.0 (45). The optimal number of genetically diverged BAPS groups (*K*) was estimated in our data by running the estimation algorithm with the prior upper bound of *K* in the range of 1 to 20. Since the algorithm is stochastic, the analysis was run in 20 replicates for each value of *K* to increase the probability of finding the posterior optimal clustering with that specific value of *K*.

On the assumption that patients were infected near their residences, the latitude and longitude coordinates of a location in the vicinity of patients’ residences at the time of the first clinical visit were collected, including for retrospective isolates, by using handheld global positioning system (GPS) devices (Garmin eTrex 20). When exact residence locations were missing, we used the latitude and longitude of the village center. QGIS v.2.14.1 (46) was used to generate the figures of the geographical distribution of Congolese M. ulcerans. The QGIS Python plugin Points displacement was used to modify point shape files, where point features with the same position overlapped. Point displacement rendered such features in a circle around the original “real” position. Geographical analysis of diversity and the overlaying of a phylogenetic tree were performed with GenGIS v2.5.0 (47), based on the household GPS coordinates of patients and whole-genome maximum likelihood (ML) phylogenies of the corresponding M. ulcerans isolates.

### Maximum likelihood phylogenetic analysis.

Maximum likelihood (ML) phylogenies were estimated ten times from SNP alignments using RAxML v8.2.4 (48) under a plain generalized time reversible (GTR) model (no rate heterogeneity) with likelihood calculation correction for ascertainment bias using the Stamatakis method (49). Identical sequences were removed before the RAxML runs. For each run, we performed 10,000 rapid bootstrap analyses to assess support for the ML phylogeny. The tree with the highest likelihood across the ten runs was selected. We used TreeCollapseCL v4 (50) to collapse nodes in the tree with bootstrap values below a set threshold of 70% to polytomies while preserving the length of the tree.

### Bayesian phylogenetic analysis.

We used BEAST2 v2.4.4 (51) to date evolutionary events, determine the substitution rate, estimate the demographic history, and produce a time tree of M. ulcerans from the Democratic Republic of the Congo. We used Path Sampling (52) as implemented in reference 11 to compare the performance of two competing coalescent demographic methods: constant size (parametric) and the extended Bayesian skyline plot (EBSP; nonparametric). The model with the EBSP tree prior had the highest marginal likelihood (Bayes factor [BF] = 10.36).

An uncorrelated log-normal relaxed molecular clock (53) was used with the EBSP demographic method and bModelTest (54) to infer a genome scale Congolese M. ulcerans time tree and with tip dates defined as the year of isolation (Table S1). BEAUti xmls were manually modified to specify the number of invariant sites in the genome. Analysis was performed in BEAST2 using a total of 5 independent chains of 800 million generations, with samples taken every 80,000 Markov chain Monte Carlo (MCMC) generations. Log files were inspected in Tracer v1.6 (55) for convergence and proper mixing and to see whether the chain length produced an effective sample size (ESS) for all parameters larger than 300, indicating sufficient sampling. LogCombiner v2.4.0 (51) was then used to combine log and tree files of the independent BEAST2 runs, after having removed a 30% burn-in from each run. Thus, parameter medians and 95% highest posterior density (HPD) intervals were estimated from 35,000 sampled MCMC generations. The analysis was also replicated on ten random subsets of 100 taxa of the complete taxon set to test if our results were affected by sampling bias. To ensure the prior parameters were not overconstraining the calculations, the entire analysis was also run while sampling only from the prior, and the resulting parameter distributions were compared in Tracer. TreeAnnotator v2.4.0 (51) was used to summarize the posterior sample of time trees in order to produce a maximum clade credibility tree with the posterior estimates of node heights visualized on it.

When using the EBSP tree prior, the mycobacterial population history is coestimated with the time tree in a single analysis. Briefly, the approach reconstructs the demographic history by taking advantage of the relationship between the population size (N) and the length of coalescent intervals (ɣ) in the estimated time-tree: N_i_ = ɣ_i_ i(i − 1)/2, where i representing the number of lineages in a particular coalescent interval (18). The result is a piecewise reconstruction of the mycobacterial population history along the time tree. The estimated timing of population increases and decreases is dependent on the estimated substitution rate. A potential source of error when estimating the substitution rate is that tip dates alone, rather than the link of tip dates associated with sequence data, might be driving the results, especially when the sequence data lack temporal phylogenetic information (56). Therefore, a permutation test was used to assess the validity of the temporal signal in the data. This was undertaken by performing 20 additional BEAST2 runs (of 800 million MCMC generations each) with identical substitutions (bModelTest), clocks (uncorrelated log-normal relaxed), and demographic models (EBSP) but with tip dates randomly reshuffled to sequences (57). This reshuffled “null set” of tip date and sequence correlations was then compared with the substitution rate estimate of the genuine tip date and sequence correlation.

### Data availability.

New Illumina short-read data for the study isolates have been deposited in the NCBI SRA under BioProject accession PRJEB4025. Both PacBio long-read data and the assembled closed chromosome sequence for the Congolese M. ulcerans strain ITM032481 (SGL03) were uploaded to ENA under study accession PRJEB30333.
