# Supplementary figures and images for: Mycobacterium ulcerans Population Genomics To Inform on the Spread of Buruli Ulcer across Central Africa
Source: mSphere. 2019 Feb 6;4(1):e00472-18. doi: 10.1128/mSphere.00472-18 (PMC6365612; doi:10.1128/mSphere.00472-18)

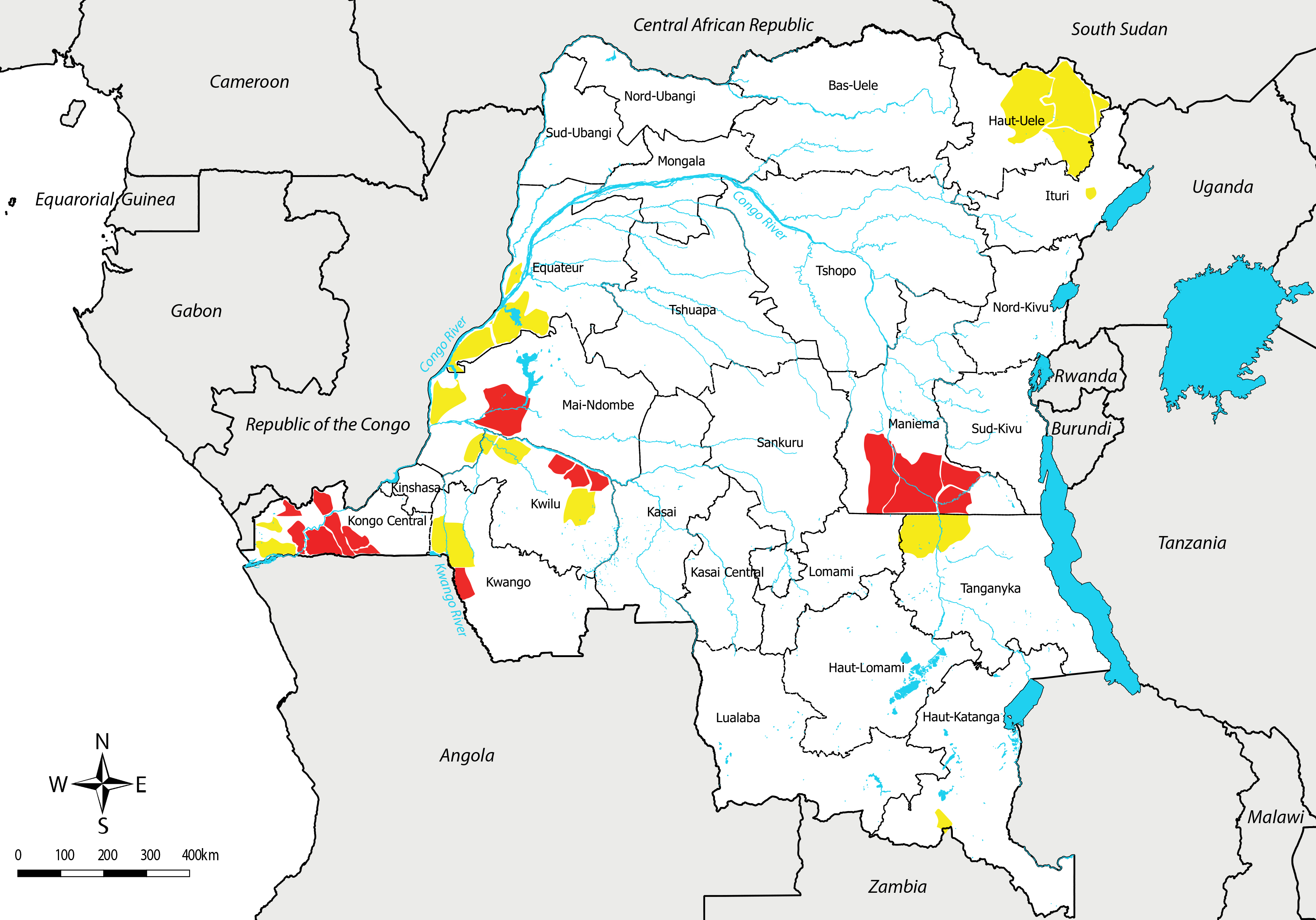

Supplement: FIG S1 [file mSphere.00472-18-sf001.jpg]

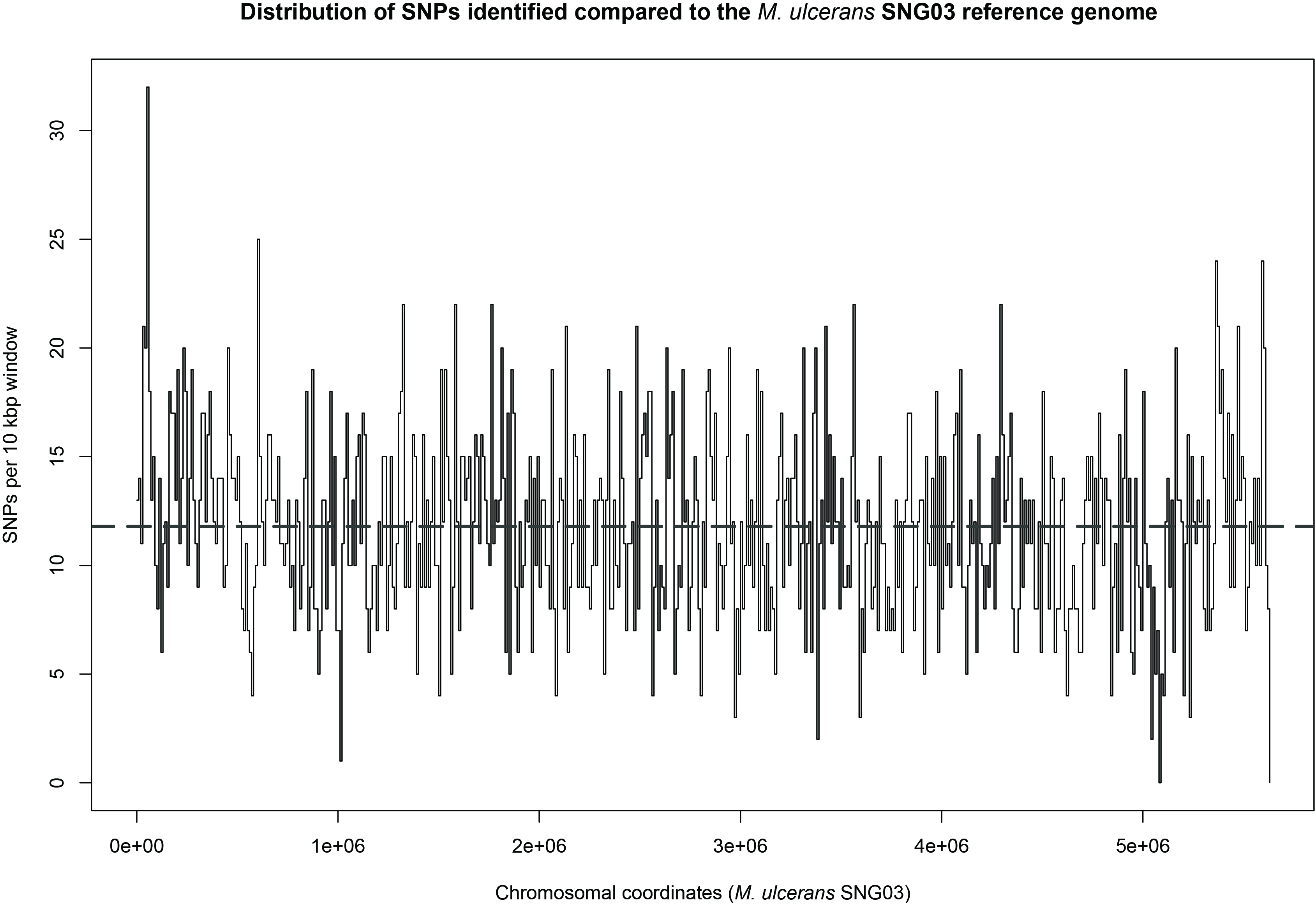

Supplement: FIG S2 [file mSphere.00472-18-sf002.tif]

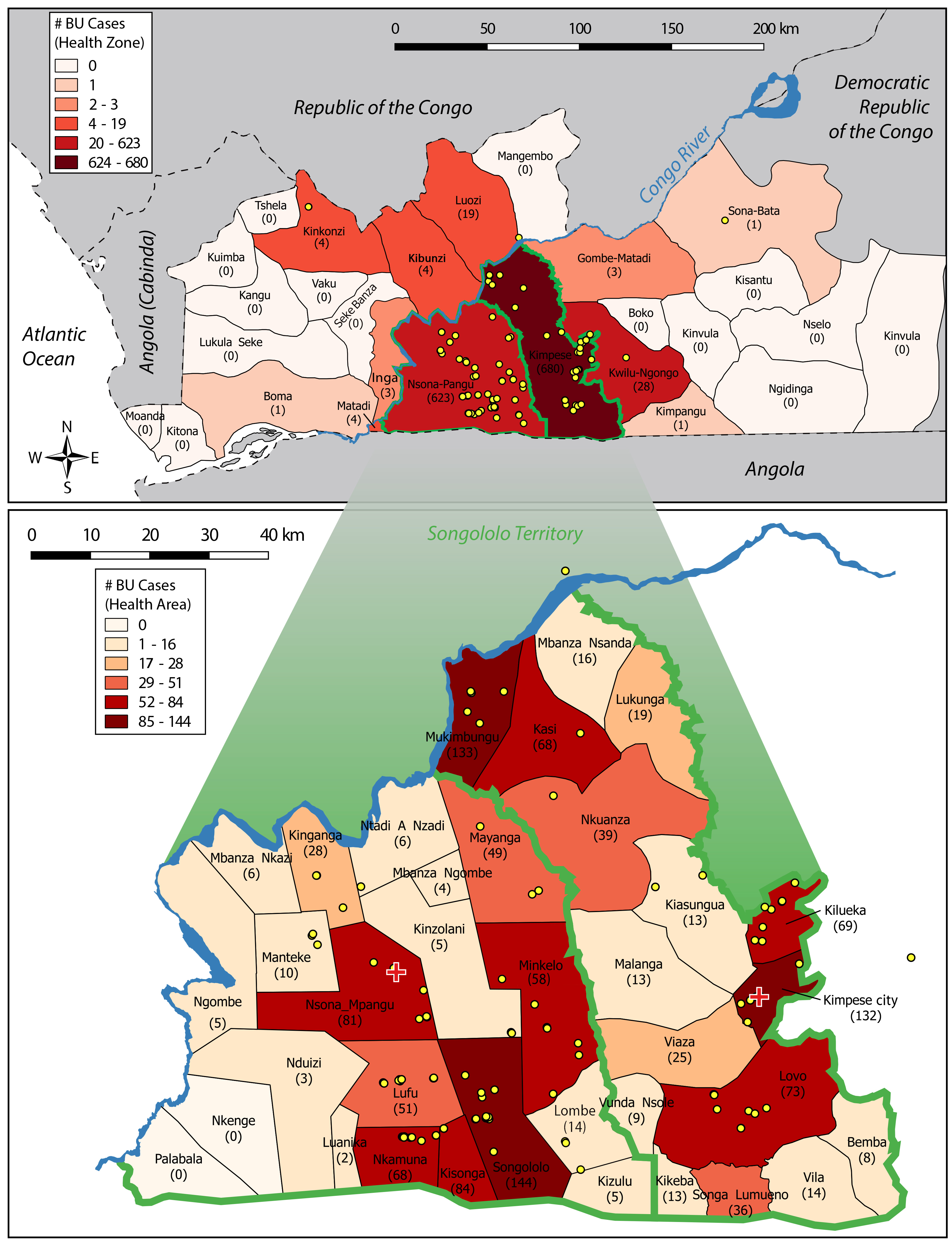

Supplement: FIG S4 [file mSphere.00472-18-sf004.jpg]

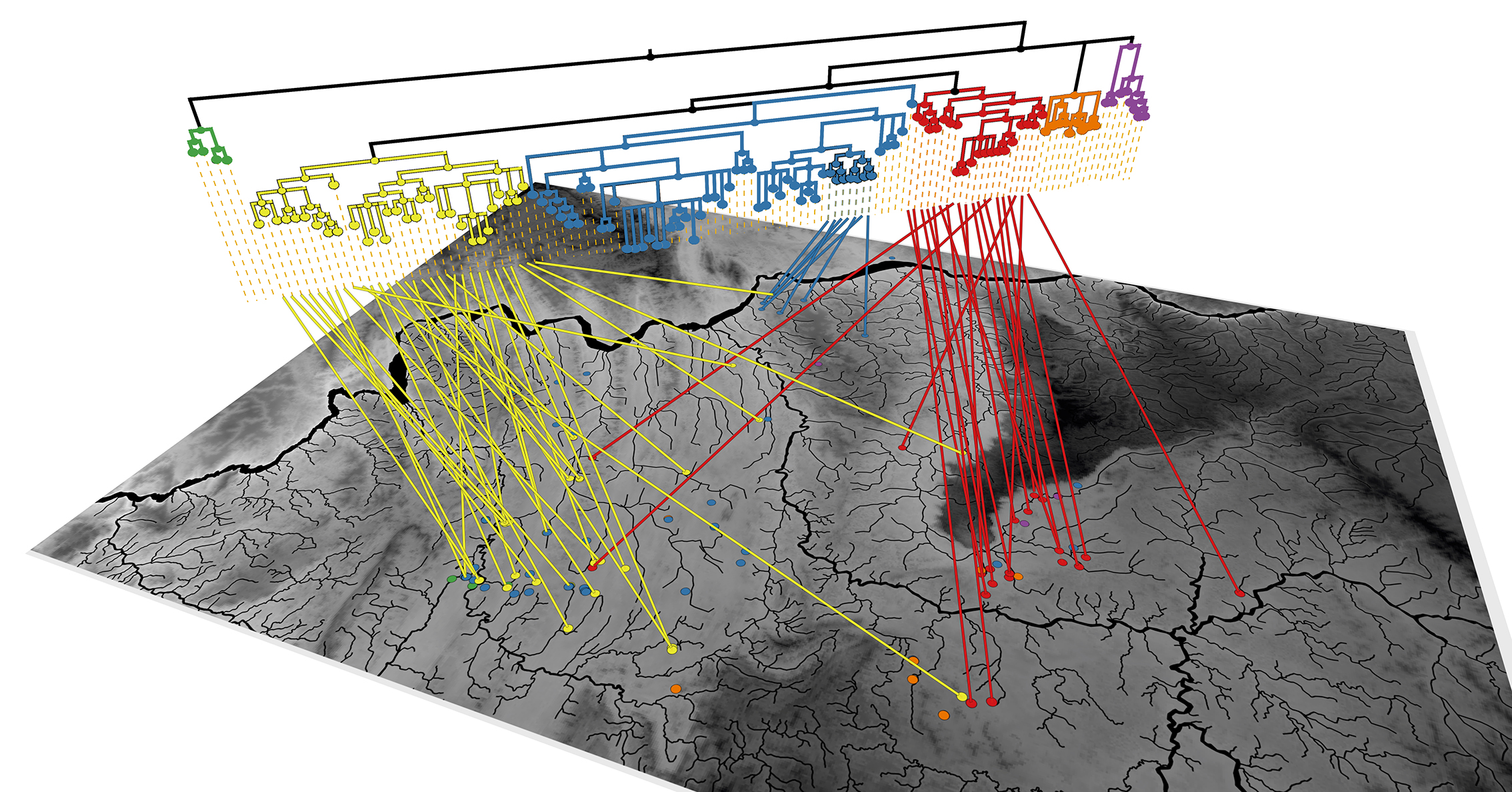

Supplement: FIG S5 [file mSphere.00472-18-sf005.jpg]

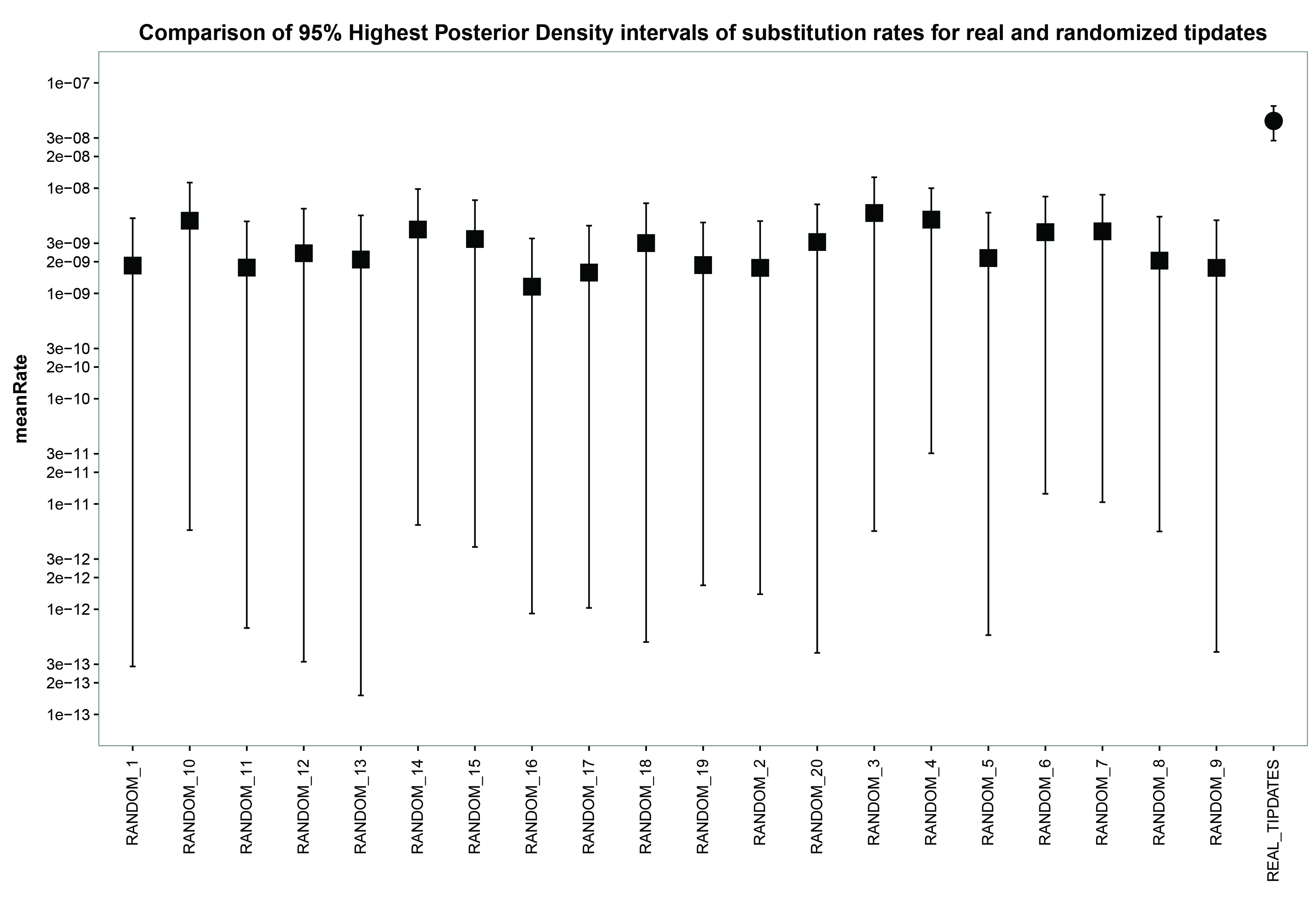

Supplement: FIG S6 [file mSphere.00472-18-sf006.tif]
